# The Inmate Who Continues to Seize: Delayed Diagnosis of Zolpidem Withdrawal Due to Functional Mimics

**DOI:** 10.7759/cureus.27231

**Published:** 2022-07-25

**Authors:** Ashish Shrivastava, Laxshika Raveendran, Yung-Tian A Gau

**Affiliations:** 1 Pediatrics, Children's Hospital of Michigan, Detroit, USA; 2 Neurology, State University of New York Upstate Medical University, Syracuse, USA; 3 Neuroscience, Johns Hopkins University, Baltimore, USA

**Keywords:** multidisciplinary management, status epilepticus, functional seizure, zolpidem withdrawal, functional neurological disorder, mental health

## Abstract

Functional neurological disorder (FND) is a constellation of common neurological symptoms without exact organic pathophysiology. The disease arises from aberrant neural computation, and its diagnosis is made upon positive clinical features. FND has emerged as a challenge to healthcare, as clinicians often have limited instructions in assessing it during their career, mainly when there are preexisting organic entities. Here we discuss an inmate whose diagnosis of zolpidem withdrawal seizure is delayed due to co-existing functional mimics and eventually led to an unfavorable outcome. We also review and summarize the current consensus on FND diagnosis and management. Together this report highlights the importance of careful investigation in atypical clinical presentation, with the intent to improve care for both organic and functional neurological patients.

## Introduction

Formerly known as conversion disorder, FND is a condition at the interface between neurology and psychiatry where individuals present with neurological complaints showing patterns of clinical features without distinct organic explanations [1,2]. The modern terminology is a neutral descriptor and acknowledges FND patients as victims of abnormal brain computation [3]. While among the most common neuropsychiatric disabilities, especially those with pre-existing organic neurological diseases, FND patients typically fall through the cracks in the health system with symptoms discounted and unmet medical needs [1,2,4]. This unfortunate scenario can be further complicated if individuals show stereotypical psychosocial characteristics, such as poor socioeconomic status or substance use disorders [5]. To raise awareness for this population, here we illustrate a case where an incarcerated male presented with a zolpidem withdrawal seizure, a known yet uncommon manifestation that was masked by his comorbid functional mimics. His severe withdrawal was neglected despite other suggestive syndromic symptoms, resulting in untoward convulsive status epilepticus (SE).

## Case presentation

R was a 24-year-old gentleman taken into the correction facility about 12 hours ago. The prison officers, during rounds, found him down in his cell, lethargic, confused, with a streak of blood coming from the corner of his mouth. R was therefore sent to our ED, where he had no recollection of the event and complained of pain in his right ankle. Orthopedic surgeons identified and managed an ankle fracture. R was then admitted to the Hospital Medicine service for post-procedural care. We also started him on levetiracetam with suspected seizures and arranged the spot electroencephalography (EEG). His labs and magnetic resonance imaging (MRI) were otherwise unremarkable.

**Figure 1:**
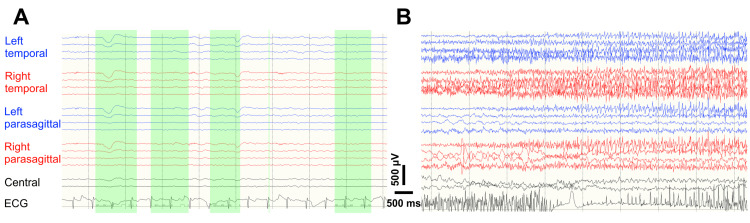
Representative EEGs (A)   The spot EEG showed no epileptiform discharges when the patient performed hyperventilation, verbally primed as a suggestive seizure maneuver here (green boxes). Meanwhile, he reported a “current” traveling upward in his right leg that was seen shaking. (B)   During the continuous EEG, the patient had several seizures with right legs jerking, rightward gaze, right arm extended, left arm postured into a figure of four, and soon after GTC. The seizures, on average, lasted for about 1 minute. This EEG demonstrated emerging rhythmic muscle artifact as he went through the GTC phase. Of note, there seemed to be semi-rhythmic 2-3 Hz discharges from bilateral parasagittal and central chains preceding the muscle artifact. GTCs: generalized tonic-clonic seizures

Within the first 24 hours, while awaiting the EEG, R had two seizure-like episodes. Staff heard him exclaiming about a “current running up the leg”, rushed to the bedside, and witnessed his leg jerking or trunk tensing. Each episode lasted about 30-60 seconds, during which he was unresponsive to external stimuli. Following each episode, he appeared confused and incoherent, requiring one to two hours to return to baseline. As a result, valproate was added. The next day the spot EEG was performed, during which he had two seizure-like spells, slightly different from his usual ones. Our neurophysiologists primed him with mental cues regarding how strong the hyperventilation was as a seizure trigger. Upon conducting the maneuver, R indeed reported the same “current-like sensation”, followed by ringing in his head, and lastly, right leg jerkiness. The phenomenon was reproducible on two separate occasions, and neither had an EEG correlation (Figure 1, Panel A). His seizure-like activity was deemed functional given how suggestible and reproducible the spells were. On the recommendation of neurophysiologists, we discontinued all anti-seizure medications and planned to send him back to the correction facility in the coming morning. 

He proceeded through the night event-free. Nevertheless, in the morning, he became hemodynamically unstable with tachycardia at 140s, tachypnea in the 30s, and hypertensive around 160s over 100s. Within the next two hours, he had three impaired awareness, focal clonus-onset - right leg jerking - to bilateral tonic-clonic seizures, associated with cyanosis and incontinence. His consciousness did not return to baseline and, because of progressive inability to protect the airway, soon developed acute respiratory failure. Consequently, he was transferred to the intensive care unit and re-loaded with an SE dose of levetiracetam. The continuous EEG captured runs of discharge when the seizures recurred (Figure 1, Panel B). We hence titrated the levetiracetam to the maximum dose and restarted valproate. After about 48 hours of intensive monitoring, R was eventually seizure-free and out of respiratory risk. However, he was still tachycardiac, tachypneic, and hypertensive without apparent causes. Upon further questioning, R revealed his zolpidem use habit (30-60 counts of 5 mg tablets daily). The last massive consumption happened right before his incarceration, about 72 hours prior. He also acknowledged alcohol withdrawal seizures in the past and had quit drinking ever since. 

Now having a seizure of zolpidem withdrawal in mind, we detoxified him with a high and brief course of diazepam (10 mg every hour for 4 hours), followed by an increased dose of valproate (500 mg three times a day) as the adjuvant. Eventually, his zolpidem withdrawal-associated hyper-autonomics resolved on the fifth day, so he was downgraded back to the floor. To address his dependence, we followed the advice of our addiction psychiatrist and maintained him on diazepam taper (10 mg four times a day, followed by a daily 5 mg decrease). On day 11, R was finally stable enough to return to the correctional center.

## Discussion

Functional seizure (FS), previously labeled as psychogenic non-epileptic seizure, is a form of paroxysmal FNDs manifesting as episodic changes in sensorimotor, autonomic, and/or responsiveness. The spells of FS mimic those of epileptic seizures superficially but do not share the fundamental physiology [6]. FSs have a prevalence of 2-33 out of 100,000 people in the general population and an incidence of 1.5-5 per 100,000 person-years, posing a universal challenge to healthcare [7]. 

While the mechanism is poorly understood, the revised terminology reflects the current understanding that FND, contrary to the previous belief, is not imagined or feigned [5]. Evidence shows that FND patients have a higher mortality rate than healthy individuals [8,9]. Moreover, compared to recruited controls purposefully feigning weakness, individuals with functional weakness exhibit distinct network differences in functional MRI, where the frontal lobes seem to modulate the motor areas [10] actively. This distinct network change indicates that the brain regions encoded the “sense of voluntariness in moving” might be dysfunctional in these patients and accountable for their functional weakness [10].

In line with the recent revision of the Diagnostic and Statistical Manual of Mental Disorders, the diagnosis of FNDs now emphasizes positive findings [11]. For example, individuals with FS typically present with marked shaking reminiscent of generalized tonic-clonic seizures (GTCs) and less frequently with isolated, focal symptoms [12]. Other features suggestive of FS include fluctuating symptomatology, ictal response to startle, extended duration of attacks, eyes shut and resistant, wild thrashing, side-to-side head movements, out-of-phase limb jerking, ictal stuttering/complex verbalization, and detailed recount of ictal events [12]. Co-existence of inter-ictal FNDs (e.g., functional weakness) or psychosocial stress (e.g., trauma, family dysfunction) can also be supportive [13]. Notably, while no longer a diagnosis by exclusion, a careful search for alternate or comorbid organic entities remains mandatory [11]. Simultaneous monitoring of clinical spells and physiology - including EEG, electromyography, electrocardiography and oxygen saturation, etc. - is the gold standard and can provide clues for exclusion (for example, ruling out epileptic seizure or convulsive syncope) [1]. When a longitudinal video EEG is unavailable, there are several alternatives (e.g., ambulatory EEG, neurohumoral, or neuroimaging) provided by the International League Against Epilepsy with different levels of diagnostic confidence; the best evidence lies in postictal prolactin, where a normal value is 89% sensitive in detecting FSs and an elevated level is seen in 88% of GTCs [1,12]. 

To manage FNDs effectively, it is essential to establish a definitive diagnosis, ensure multidisciplinary, longitudinal care, and make an individualized treatment plan [14]. To start with, clinicians, preferably a neurologist, should introduce the FND diagnosis to the patients, clarify the rationale, validate the debility and, last but not least, stop the non-indicated treatment (for example, empirical anti-seizure medications) [15]. Successful diagnosis delivery can relieve patients and facilitate the next step of care, which is individualized, multidisciplinary, and longitudinal management [1,14]. Studies demonstrate that multidisciplinary involvement (including neurologist, psychiatrist, physiotherapist, and primary care provider) significantly alleviates the symptoms and reduces hospital readmissions [14]. Individualized cognitive behavioral therapy significantly reduces seizure frequency and more seizure-free time [16]. Likewise, goal-directed physiotherapy should focus on meaningful activities for the patients (e.g., walking or running) instead of on the affected body parts (e.g., strengthening of muscle groups) [17]. This tailored approach can improve functional symptoms by 72% compared to 18% in the traditionally managed group [17]. Since successful FND management is based on a strong therapeutic alliance, researchers found that primary care providers, as part of the multidisciplinary team, can act as the main resource for workup, medication, and financial or social paperwork to minimize inconsistencies and ensure longitudinal, coordinated care [14]. 

Risk factors of FNDs include substance use, mental disorders, and existing neurological diseases [2,18]. It is not uncommon for individuals with organic neurological diseases to develop FNDs, leading to a caveat in timely diagnosis and management [2]. FSs are one of the main neuropsychiatric issues associated with epilepsy [7]. Out of the entire US epilepsy population, 10-20% have the comorbidity of FSs with relatively higher complication rates, a phenomenon which can be partially attributed to vigilance decrement from the providers [7,14,18]. The relative inattentiveness is further complicated in a rare scenario, for instance, in our zolpidem withdrawal seizure patient. Traditionally, zolpidem is believed to be less habit-forming, and demonstrated in several trials that abrupt discontinuation did not result in withdrawal [19,20]. In practice, the most common symptoms for the 1-10% zolpidem use population that makes experience withdrawal are mild anxiety, restlessness, or moderate sympathetic activations [19]. The most severe seizures occur in less than 1% of the withdrawal patients (thus estimated as 0.01-0.1% of zolpidem use) [19]. The rare nature of zolpidem withdrawal seizure and the reduced vigilance together resulted in the unfortunate consequence here.

While it is well-perceived that simultaneous recording of typical events on video and EEG is the gold standard for diagnosing isolated FSs [15], we still prematurely aborted the necessary workup in our case. It was not until he had serious complications that the continuous EEG was pursued, and the appropriate diagnosis was achieved. In retrospect, we did not record his typical episode during the first spot EEG. Second, the electrical field of “current-like sensation and jerkiness in one leg” is highly localized and can be missed with a short EEG; therefore, its lack of EEG correlate usually deemed inconclusive when diagnosing FSs [1,4]. Thirdly, suggestive seizure inductions, such as the verbal priming for our case, raise ethical concerns and are relatively discouraged nowadays [1]. With 17-22% of FS patients having concurrent epilepsy, this case reminds us of the standard caveat of coexisting organic and functional etiologies and emphasizes the need for careful investigations before attributing all the symptoms to FNDs [6].

## Conclusions

This report illustrates an inmate’s delayed diagnosis of zolpidem withdrawal because of our insufficient understanding of FND/FSs. The delay ultimately led to SE evolution and intensive care requirement that could have been avoided. Through this unfortunate incidence, we hope to raise clinical awareness of FNDs, specifically in the context of atypical presentations and comorbid organic diseases. Furthermore, we review the current consensus and highlight the significance of multidisciplinary, individualized, and longitudinal management in this particular population.
